# Supplementary material for: Differential Functional Contribution of BK Channel Subunits to Aldosterone-Induced Channel Activation in Vascular Smooth Muscle and Eventual Cerebral Artery Dilation
Source: Int J Mol Sci. 2023 May 12;24(10):8704. doi: 10.3390/ijms24108704 (PMC10218309; doi:10.3390/ijms24108704)
Supplement: Supplementary file 1 [file ijms-24-08704-s001.zip › ijms-2283693-supplementary.pdf]

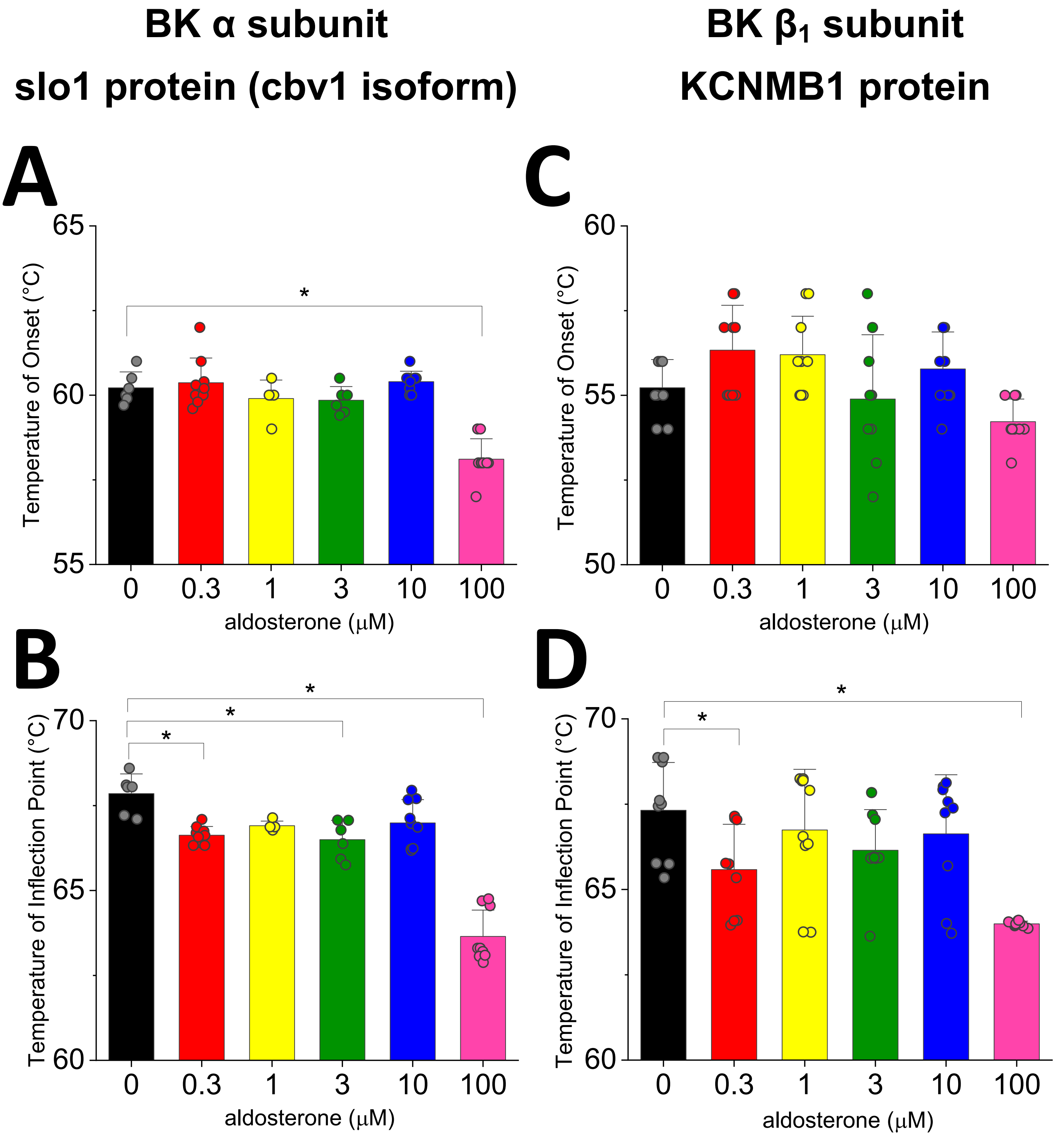


**Fig. S1: Onset and Inflection point temperatures of slo1 and KCNMB1 protein thermal unfolding in presence of various aldosterone concentrations. A.** Bar graph depicting that the onset of protein unfolding for the CBV1 isoform differed between vehicle control (0 μM aldosterone) and 100 μM aldosterone. **B.** Bar graph depicting that the temperature of inflection point for the CBV1 isoform differed from vehicle control at 0.3 μM, 3 μM, and 100 μM aldosterone. **C.** Bar graph showing lack of changes in temperatures of onset for KCNMB1 protein unfolding in presence of different aldosterone concentrations. **D.** Bar graph depicting that the temperature of inflection point for KCNMB1 protein differed from vehicle control at 0.3 μM and 100 μM aldosterone.

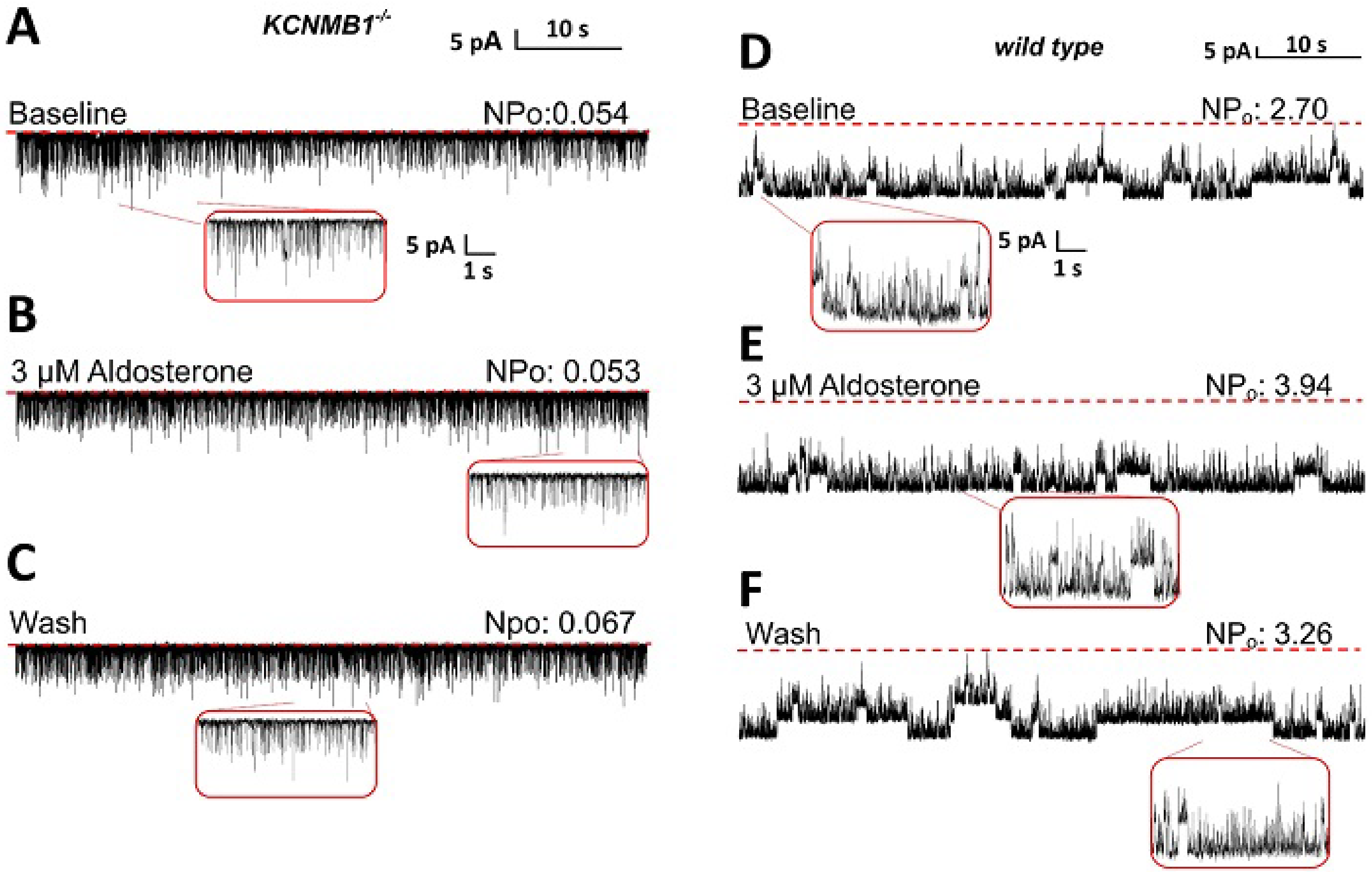


**Fig. S2.** Aldosterone activates smooth muscle BK channels from *wt* mouse middle cerebral artery (MCA) in a concentration-dependent manner; this action is blunted in myocyte BK channels from *KCNMB1*$^{-/-}$ mice. On the left: channel activity traces before (**A**) during (**B**) and after (**C**) application of 3 µM aldosterone to the cytosolic side of inside-out (I/O) patches from *KCNMB1*$^{-/-}$ myocytes. Channel steady-state activity (NPo) as function of aldosterone concentration shows mild increase only at 100 µM. On the right: channel activity traces before (**D**) during (**E**) and after (**F**) application of 3 µM aldosterone to the cytosolic side of I/O patches from *wt* myocytes. In wt cells, steroid action is concentration-dependent: $EC_{50}$=3 µM; $EC_{MAX}$=10 µM. Red dotted lines indicate the baseline (all channels closed). Time-expanded traces are shown within red boxes.

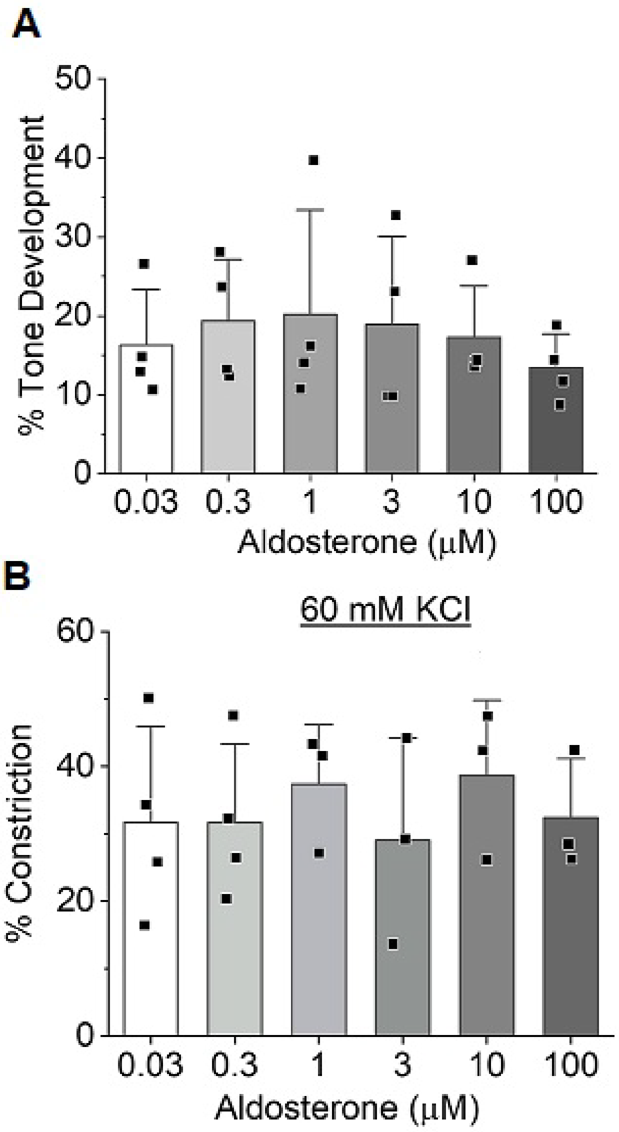


**Fig. S3. A.** All mouse middle cerebral artery (MCA) segments probed with different concentrations of aldosterone were able to develop myogenic tone after pressurization to 60 mmHg, matching that of previous publications (*Bukiya et al., 2008; 2009; North et al., 2018; 2020*). **B.** After exposure to aldosterone, all MCA segments constricted in response to depolarizing, 60 mM KCl, as previously documented in this preparation by *North et al.* (2020).
